# Using the Behaviour Change Wheel to design an intervention for partner abusive men in drug and alcohol treatment

**DOI:** 10.1186/s40814-021-00911-2

**Published:** 2021-10-28

**Authors:** Elizabeth Gilchrist, Amy Johnson, Mary McMurran, Danielle Stephens-Lewis, Sara Kirkpatrick, Benjamin Gardner, Caroline Easton, Gail Gilchrist

**Affiliations:** 1 University of Edinburgh, Edinburgh, UK; 2 Lancaster, UK; 3 Gloucestershire University, Cheltenham, UK; 4 Welsh Women’s Aid, Cardiff, UK; 5 King’s College London, London, UK; 6 Rochester Institute of Technology, Rochester, USA; 7 Yale Psychiatry, New Haven, USA

**Keywords:** Substance use, Intimate partner abuse, COM-B, Group intervention, Feasibility, Intervention development

## Abstract

**Background:**

We aimed to establish what core elements were required in a group therapy programme for men who disclose perpetrating intimate partner abuse in a substance use setting and develop, and test the feasibility of delivering an intervention in this setting.

**Methods:**

We describe the theoretical development and feasibility testing of an integrated substance use and intimate partner abuse intervention (‘ADVANCE’) for delivery in substance use services. We employed a comprehensive eight-stage process to guide this development applying the ‘COM-B’ (‘capability’, ‘opportunity’, ‘motivation’ and ‘behaviour’) model for intervention design which specifies the following: (1) define the problem, (2) select the target behaviour, (3) specify the target behaviour, (4) identify what needs to change, (5) identify intervention functions, (6) identify policy categories, (7) select behaviour change techniques, and (8) design a mode of delivery. The development was informed by primary research conducted by the authors, consulting with organisation steering groups and by those with personal experiences. The identified targets for intervention and mode and method of delivery were then refined over 4 intervention development meetings, using the nominal group technique with the ADVANCE experts, then further refined following consultation with service user groups and wider expert groups via a learning alliance meetings.

**Results:**

Our final intervention, the ADVANCE intervention consisted of a group intervention comprising of up to four pre-group individual interviews, followed by 12 × 2-h group sessions supported by integrated safety work for victim/survivors, and risk and safety support and integrity support for the professionals. The main targets for change were personal goal planning, self-regulation, and attitudes and beliefs supporting intimate partner abuse. The intervention was regarded as very acceptable to both staff and clients in substance use services, with group attendees reported positive behaviour changes and development of new skills.

**Conclusion:**

We have demonstrated the ability to employ a structured eight-step process to develop an integrated intervention to address substance use-related intimate partner abuse that is acceptable to staff and clients in substance use services. This led to a feasibility study (ISRCTN 79435190) involving 104 men and 30 staff at three different locations across the UK was conducted to assess the feasibility and acceptability of the intervention and to refine the content and approach to delivery (BMC Public Health, 21: 980, 2021).

## Introduction

### Background

Intimate partner abuse (IPA) or violence (IPV)^1^ refers to any behaviour within an intimate relationship that causes physical, psychological, or sexual harm, and includes not only physical violence but also emotional and psychological abuse and controlling behaviours [70]. IPA is a prevalent global public health problem with severe consequences for victims and family members exposed to the violence and abuse [64]. While men can be victims, IPA disproportionately affects women, with 30% globally reporting lifetime IPA victimisation [70]. Victims experience physical and mental health problems and family members, particularly children, experience adverse health, social, and developmental effects [33].

Many factors are associated with the perpetration of IPA [21] with the consensus that there is no single factor that explains why some men may perpetrate IPA and not others. These factors impact at individual, community, and systemic levels [60]. Table 1 provides a summary.

**Table 1:** Factors affecting likelihood of IPA perpetration (this is a synthesis of factors identified in recent, relevant international reviews [1, 25, 31, 54, 61, 66])

Level of Influence	Factors increasing IPA
Cultural	Patriarchy Economic inequalities Honour culture
Demographic	Young age Male sex Low socioeconomic status Challenges of acculturation (competing cultures)
Neighbourhood/community	Collective efficacy Low social cohesion/control Neighbourhood disorder/disadvantage Alcohol outlet density Involvement in drinking/drug taking subculture
Family	Experience of child abuse and neglect IPA exposure in childhood
Peer Association/influence	Negative /antisocial/substance using peers Lack of social support Lack of emotional support
Relationship	Unstable/unequal relationship status Satisfaction Relationship conflict Jealousy
Psychological/behavioural	Insecure/dismissive attachment Negative emotionality Anger Impulsivity Low mood Anxiety Substance use Personality disorder(s): antisocial/borderline Low self-esteem Suicidality
Cognitive	Hostile beliefs Hostile attitudes Hostile attributions Entitlement Rigid sex roles

For the purposes of this study, the focus is primarily on individual and relationship factors, understood against the background of gendered violence, perpetuated by systems of patriarchal power [15, 51]. Individual factors include personality traits, witnessing violence between parents or experiencing physical violence from parents in childhood [7, 30, 68], beliefs supportive of IPA, and mental health problems [42, 69]. Relationship factors [12] include the status of the relationship (e.g. marital or co-habiting), relationship conflict, parenting disagreements, and general stressors linked to factors such as money, housing, and employment. Relationship factors can also include the partner’s personality traits, substance use status, and history of violence victimisation [54, 65]. These factors can operate independently, summatively, or interactively to increase the likelihood of IPA.

#### Interventions addressing IPA

Interventions to reduce IPA include prevention, identification, victim support and safeguarding, and perpetrator programmes [66]. These approaches can effectively occur together, but the focus here is specifically on interventions for male perpetrators of IPA against women. Current data continues to disproportionately highlight that women are affected by male perpetration of IPA [70]. Perpetrator programmes can be effective in reducing IPA [31, 49], yet no single approach can be definitively supported [1, 49, 61]. There is evidence for a range of approaches with different theoretical standpoints: feminist-based Duluth model group treatments [32], cognitive-behavioural interventions, motivational interviewing [49, 59, 61, 66] and motivational enhancement [41] or individual motivational input [37]. Additionally, there is evidence supporting holistic, psychoeducational, risk-needs-responsivity and couple counselling models [10, 34]. However, issues have been highlighted for these approaches, particularly when reviewing recidivism rates (see [61] for further discussion on the varied approaches). There is also evidence that adapting or enhancing ‘batterer intervention programs’ can add to effectiveness: these can be adaptations that include an element of restorative justice [50] or cultural and language enhancement [18]. Evidence suggests considering adverse childhood experiences [19] in the perpetrator population [28] and incorporating a ‘trauma-focus’ within the interventions might be of value [67], however, as yet there has been limited evaluation of trauma-informed approaches to IPA treatment. There has also been some debate about whether there are significant differences between court mandated and ‘voluntary’ programmes and the effectiveness of both [1]. Motivation to engage is important, and coercion into treatment is not the most effective strategy [45]; however, clinical knowledge suggests that many participants in both types of programme, both court-mandated programmes and ‘voluntary’, are initially extrinsically motivated. This can be either to avoid more serious justice sanctions, or to achieve another goal, e.g. having been referred by a partner to prevent them for leaving (sometimes referred to as ‘partner referred’) or to gain positive evaluation in relation to some other process, for example, to gain a more positive evaluation as a parent in the context of proceedings relating to child protection or child contact. Interventions are typically delivered by social services, criminal justice services, and third sector organisations. One gap is the delivery of perpetrator interventions in substance use services, which an important omission is given the impact of substance use (SU) on IPA.

#### The interplay between SU and IPA

While substance use has been viewed as an excuse for IPA perpetration [23], there has been increasing acknowledgement of the role of substance use as an aggravating factor [36] or a risk factor for IPA [44]. Meta-analyses have shown significant reductions in violence for men in substance use treatment services [31, 49] and subgroup analysis has indicated that treating substance use in perpetrator programmes potentially enhances outcomes [31]. Recent research has increased the awareness of the association between substance use and the risk of IPA incidence and level of injury [7, 8, 35, 71]. In addition to higher rates of substance use among women who have experienced IPA victimisation [9, 17, 62], IPA perpetration is more prevalent among men in substance use treatment when compared with the general population [16, 26]. Despite this, few men attending substance use treatment who indicate IPA perpetration report having ever received support for their violent and controlling behaviour [6, 27].

Stephens-Lewis et al. [61] conducted a systematic review and meta-analysis of the effectiveness of perpetrator programmes for men who use substances. The review identified few trials (*n*=9), and of these, five trials were integrated IPA and substance use programmes. The meta-analysis within this review showed no difference in substance use (three trials) or IPA outcomes (four trials) compared to substance use treatment as usual. However, the small number of studies along with the heterogeneity of these means that it is premature to conclude that integrated interventions do not work. These trials’ results do prompt further questions about (1) the theory, content, mode of delivery, and duration and intensity of interventions; (2) the characteristics of the individuals requiring treatment, including the types of substances used, the type of abuse perpetrated, and the nature of the relationship between substance use and IPA perpetration; and (3) what outcomes are assessed, where information is sourced, and the duration of follow-up.

In beginning to address these questions, Gilchrist et al. [25] conducted a meta-ethnography of qualitative studies to explore how substance use features in survivors’ and perpetrators’ accounts of IPA. The themes identified related to the complex interplay between substance use and IPA in the context of intoxication, withdrawal and addiction, the impact on relationships, wider dynamics of power and control, and psychological vulnerabilities. Survivors were more likely to see substance-related IPA as part of a pattern of abusive behaviour, whereas perpetrators tended to describe a causal relationship between intoxication and discrete incidents of IPA perpetration. Irritability and frustration during withdrawal from or craving for alcohol or drugs, and/or a partner’s refusal or failure to obtain money for alcohol or drugs increased the likelihood of violence. Survivors were more likely to identify abuse being related to substance use, and to focus on how substances impacted their relationship and dynamics of power and control. Perpetrators perceive a change in self, which enables their abusive behaviour. Behaviour change interventions need to reframe such narratives so that perpetrators take accountability for the abuse. This is key to enhancing self-responsibility and thus a willingness to change. These findings highlight the complex interplay between psycho-pharmacological effects of substances, gendered power relations and controlling behaviours that behavioural interventions should address.

Building on this, interviews were conducted with intimate partner (current and/or ex) dyads, where men were receiving treatment for substance use and who reported IPA perpetration [22, 55]. Similar to the meta-ethnography, analysis found that ‘the psychopharmacological effects of substance use (including intoxication, craving, and withdrawal) were rarely the only explanation offered for IPA’ but often intensified conflicts. Female partners of men in treatment for substance use described experiencing patterns of abusive behaviour often associated with their partner being intoxicated, craving or withdrawing from substances, including conflict resulting from the need to raising funds for substances. More specifically, substances can be independently implicated in the perpetration of coercive control, in that perpetrators may control their partners by increasing their substance dependency or restricting access to substances. This may continue to entrap women within an abusive relationship. Hence, there is a complex relationship between substance use and IPA, with an interplay between intoxication, acquiring substances, craving, withdrawal, gender power relations, and control, all of which should be considered when designing perpetrator programmes.

There were several implications from this research. There is limited evidence of effective interventions targeting substance using men who perpetrate IPA. There is also a need to recognise a number of factors that correlate with substance use and IPA. There is a need for tailored interventions that address the complex ways that substance use and IPA perpetration intersect in relation to social, psychological, and environmental factors. While power and control are implicit in understanding IPA perpetration, interventions for those men within substance use treatment should also address key risk areas, including intoxication, anger, trauma, grief, dependency [22], and the presence of mental health issues such as anxiety and depression [35]. Interventions should be tailored and use personal goals to address individual need. Integrated interventions for SU/IPA should also address intoxication, craving, withdrawal, acquisition of the substance using lifestyle, the intricate interdependencies within substance using relationships, and the gendered power dynamics underpinning substance use and IPA.

#### The ADVANCE intervention

The ADVANCE intervention, funded by the UK National Institute for Health Research, aimed to fill a gap in perpetrator programmes by developing and evaluating an integrated substance use and IPA perpetrator programme specifically for men in substance use treatment services whose substance use and IPA are associated and interconnected. This new voluntary (i.e. non-court mandated) programme aims to reduce or stop men’s IPA through a focus on both substance use and IPA and on their interconnection exclusively for this client group, who have not presented specifically for help with IPA. The underpinning belief within the ADVANCE programme is that the programme will support positive change irrespective as to whether participants are extrinsically motived to start. Whatever the initial motivation is, we believe that by setting personal goals, promoting enlightened self-interest and offering positive incentives, this can support positive change and may develop intrinsic motivation within the group. This paper sets out how the ADVANCE intervention was developed from the evidence base and literature. The development of the intervention was guided by an eight-stage intervention design process. Hoddinott [29] noted the importance of intervention development studies, namely studies that describe ‘the rationale, decision making processes, methods and findings which occur between the idea or inception of an intervention until it is ready for formal feasibility, pilot or efficacy testing prior to a full trial or evaluation’ (p. 1). A description of the design and development of the intervention facilitates understanding of the intervention and how the components contribute to the whole, thereby enhancing implementation according to the underlying rationale and paving the way for future quality improvement by elucidating aspects that may need to be changed. O’Cathain et al. [53] have identified a range of approaches to intervention development. A theory and evidence-based approach was the principal method used in ADVANCE, using the Behaviour Change Wheel.

#### Behaviour Change Wheel (BCW)

**Fig. 1:**
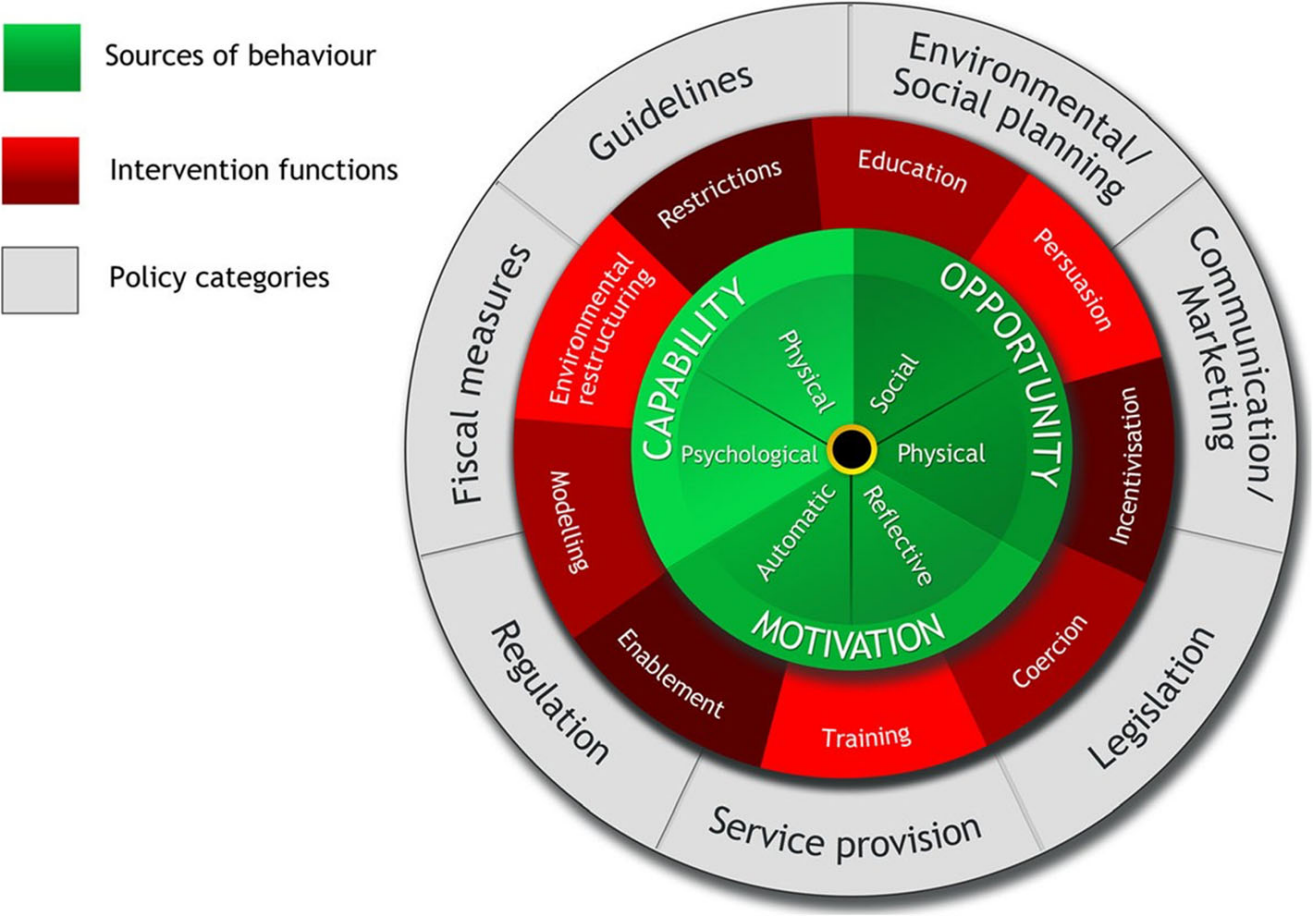
Behaviour Change Wheel (reproduced with permission of the authors) [48]

The BCW was developed to identify the type of intervention that would be the most appropriate to effect the desired change in a particular domain [48]. Underpinning the BCW is the COM-B model of behaviour, the initials of which stand for capability (C), opportunity (O), motivation (M), and the behavioural outcome (B) (see Fig. 1). This concentric model identifies a number of domains that influence behaviour, integrates and maps out the drivers of behaviour, and links these with intervention functions and policy strategies. The intervention functions include education, persuasion, incentivisation, coercion, training, restriction, environmental restructuring, modelling, and enablement. The model is a helpful tool for the development of rigorously designed interventions prior to controlled trials [47].

The COM-B model can be further elaborated using the Theoretical Domains Framework (TDF), which subdivides the COM-B components in a clinically useful way by specifically identifying 14 domains that drive behaviour [11]. These are knowledge; skills; memory, attention, and decision processes; behavioural regulation; social/professional role and identity; beliefs about capabilities; optimism; beliefs about consequences; intentions; goals; reinforcement; emotion; environmental context and resources; and social influences [46]. The BCW, based upon the COM-B and TDF, sets out a comprehensive eight-stage process for intervention design: (1) define the problem, (2) select the target behaviour, (3) specify the target behaviour, (4) identify what needs to change, (5) identify intervention functions, (6) identify policy categories, (7) select behaviour change techniques, and (8) design a mode of delivery.

This paper describes the theoretical and practical development of an integrated IPA and substance use intervention for male IPA perpetrators in substance use services. The process through which the intervention was developed will be described. The implementation of the resultant ADVANCE intervention to check on viability in practice will be reported briefly.

## Materials and methods

### Aim, design, and setting

This study reports the rationale, decision-making processes, methods, and findings which were used to develop a theory and evidence-based intervention for perpetrators of IPA attending substance use treatment—the ADVANCE intervention.

### Materials and processes

We based the development of the ADVANCE intervention on existing research and systematic reviews conducted by the authors including a systematic review of IPA interventions for perpetrators who used substances [61], a meta-ethnography of qualitative studies investigating the role of substance use and IPA [25], and interviews with couple dyads (i.e. perpetrators and victims of IPA) [22, 55].

In order to translate the current evidence related to both IPA and substance use into an integrated approach and guide the intervention design, a multi-disciplinary group came together and followed the steps of the BCW [47]. The team members came from psychology, addiction, public health, IPA, and behaviour change backgrounds. The design team and key stakeholders developed materials collaboratively over several months. Additionally, Learning Alliance groups (consisting of professionals and academics) and a Public and Patient Involvement Group (PPI) were constituted and consulted about its development at key points; their continued feedback informed the process.

Thus, the method was consultation and consensus, with the core team discussing research and practice, drafting proposals which were then taken to Learning Alliances and PPI Groups for discussion which was fed back to the core group, and amendments were made accordingly and the final programme agreed by consensus. Nominal Group Techniques (NGT) were used with the two Learning Alliances (total of 20 key stakeholders) to reach consensus on the intervention targets and content [40]. During these small group discussions, information was presented, then members were asked to write down their thoughts independently, then every member shared their feedback to the group which were recorded on a flipchart. These were then discussed and prioritised until consensus was reached on the goals for treatment and mechanisms by which change could be achieved. The main ideas were incorporated to strengthen the intervention, safeguarding, and engagement including making it more trauma informed, making gender roles and beliefs more explicit throughout all sessions, ensuring regular case management between the substance use service and the integrated safety service, and that the facilitator should follow up men who missed a session to encourage them to re-engage and also to ‘check-in’ after the sensitive session on the impact of their behaviour on their children.

People with lived experience were also consulted in small PPI groups (3 women and 10 men). They advised about the structure, approach and content of the intervention, as well as suggestions for enhancing engagement and retention. Their feedback was also incorporated including the need for the intervention to be as interactive as possible and the need to establish ground rules around confidentiality and to challenge men’s privilege/gender stereotypical beliefs about roles and norms. They also suggested the provision of a break out space with staff available to support if men wish to leave a group session for whatever reason and highlighted the importance of trying to re-engage men who had not attended a session and catching them up on what they had missed before the next session to encourage attendance.

In accordance with the BCW, we followed an eight-stage process for intervention design: (1) define the problem, (2) select the target behaviour, (3) specify the target behaviour, (4) identify what needs to change, (5) identify intervention functions, (6) identify policy categories, (7) select behaviour change techniques, and (8) design a mode of delivery. The ‘Results’ section describes these steps.

## Results

Each of the steps of intervention development is described below.

### Step 1: Define the problem

As we have shown, there is a lack of targeted effective interventions to reduce or stop IPA among male perpetrators receiving treatment for substance use. Perpetrator interventions do not address the complex role that substance use plays in IPA perpetration. The target population was men attending community services for drug and/or alcohol use in England.

### Step 2: Select the target behaviour

The aim is to reduce or stop men’s perpetration of IPA in the context of substance use.

The team members’ previous research identified a lack of understanding by perpetrators of the range of ways that substance use could impact on their IPA behaviour, and a reluctance to acknowledge the impact of their abusive behaviour on others [22, 56]. Our research also identified that there was a simplistic understanding of the impact of substances on them, often linked to simple intoxication and discussion of pharmacological effects rather than an acknowledgement of the impact of lifestyle, withdrawal, need, or intoxication and how these related to beliefs around the right to control partners [22, 56]. Therefore, reducing, or quitting substance use is important, but in pursuit of this conveying a nuanced understanding of the complex role of substance use on IPA is vital. Participants in the intervention may require a fuller understanding of the rationale for changing substance use and the positive impact on their relationship of doing so; consequently, it can be expected that their motivation to change will be enhanced. Positive impacts on other aspects of life are likely from reducing substance use, including improved physical and mental health, financial benefits, and fewer life crises. In summary, ADVANCE targets men’s IPA in the context of substance use by addressing the following three areas: (1) promoting respectful egalitarian behaviours, (2) promoting alternatives to violent and aggressive behaviour, and (3) reducing substance use.

### Step 3: Specify the target behaviour

In specifying the target behaviour, the BCW guidebook recommends consideration of who, what, when, where, and with whom ([48], p189). We would add ‘why’ as also being necessary as by understanding the function of the behaviour it may become easier to understand how to change it. The target behaviour therefore would be for male substance users who have perpetrated IPA (who), to cease IPA (what), at all times (when), in all relationship settings (where), against intimate partners (whom) in the context of substance use (why). Subsequently the three main target areas to address all issues were identified as being:

1. Promoting respectful egalitarian behaviours

- Identify the function of abusive behaviours within relationships
- Identify alternative goals and methods of achieving them for each man
- Focus on control of self, not control of others

(2) Promoting alternatives to violent and aggressive behaviour

- Increase distress tolerance: in crisis and generally
- Increase recognition of negative mood and internal triggers
- Promote emotional self-regulation

(3) Reducing substance use

- Increase awareness of personal function of substance use
- Increase awareness of the relationship between substance use and IPA
- Plan to avoid IPA risk related to substance: acquisition, intoxication, withdrawal

### Step 4: Identify what needs to change

The ADVANCE intervention proposes that change is facilitated by increasing understanding of the function of aggressive and abusive behaviours and the contribution made by substance use and gendered attitudes. The intervention promotes motivation by increasing participants’ recognition of areas that need to change and increasing participants’ capabilities by introducing skills for change. ADVANCE aims to highlight individual risks for IPA, including substance use, poor emotional regulation, and poor stress-coping, and teach participants how to reduce these risks by promoting self-regulation, and personal goal setting.

Self-regulation refers to an individual’s ability to alter a response or override a thought, feeling, or impulse [3–5]. Self-regulatory deficits have demonstrated promise in promoting abstinence from the hazardous use of substances [52]. In terms of IPA, dips in self-regulation have been indicative of perpetration [20]. Also, the ability to inhibit an impulse towards abusive behaviour in the context of intimate partner abuse is affected by substances, as highlighted in the multiple thresholds model. This model posits that substances change the balance between ‘instigating and inhibiting’ factors. People affected by substances focus more on cues that instigate abuse and are less able to inhibit abuse [35]. Strengthening the ability to read environmental cues accurately, avoid misreading natural cues as aggressive, and manage the impulse to abuse, even when affected by substances, is indicated.

Personal goal planning using SMART goal setting (this model, drawn from business, encourages precision in planning requiring aspirations to be specific, measurable, achievable, relevant, and time-limited) [38] is used in the ADVANCE model to enhance task completion by making all goals personal, explicit, and specific. These goals address reduction in substance use as well as building positive relationships and healthy lifestyles. Personal goals that are SMART, along with self-regulation enhance engagement and self-efficacy.

### Step 5: Identify intervention functions

Derived from the analysis of risk factors (Table 1) and potential intervention targets, Table 2 outlines the intervention functions for men in substance use treatment who perpetrate IPA. The nine intervention functions which we describe the development of ADVANCE are education (knowledge), persuasion (increasing desire), incentivisation (rewarding), coercion (increasing potential negative consequences), training (skills), restriction (rules/laws to prohibit undesired behaviour or promote desired behaviour), environmental restructuring (physical changes to facilitate desired behaviour), modelling (demonstrating), and enablement (removing barriers to facilitate positive behaviours).

**Table 2:** intervention components

Factors underpinning IPA in substance using men	COM-B	TDF	Intervention function	BCTs	Translation of BCTs within ADVANCE
1. Respect					
Sees himself as the victim Sees himself as unable to make positive changes Believes he will lose male identity if he changes Not believe he has choices	Reflective Motivation	Social Role/Identity Belief about capability Intentions Goals Optimism	Persuasion Education Enablement Modelling Incentivisation	Acknowledge backgrounds Promote positive images Promote self-efficacy Reward positive choices Reward attendance	Information on intergenerational IPA links Boost positive models non-abusing male Work with clients’ strengths Reward positive engagement/attendance: attend as you can Pay incentives rewarding attendance—tied to positive non-abusing goals
Lack understanding of the impact of his behaviour on partner	Reflective Motivation	Belief about consequences	Education Modelling	Demonstrate positive and negative interactions on film	Film demonstrating impact of substance led or controlling (‘protective’) behaviour on partners
2. Self-regulation					
Poor behaviour management Poor self-regulation	Physical capability	Behavioural Regulation	Enablement Modelling Incentivisation	Introduce self-management Manage Emotions SMART goals	Films demonstrating how to do it differently Demonstrate distress tolerance Incentives for Attendance
3. Substance use					
See substances as controlling his behaviour Fail to inhibit controlling or abusive behaviours	Physical Capability	Physical Skills	Education Enablement	Feedback on substances and behavioural choice Promote behaviour management Distress tolerance	Provide input on range of influences of substances on behaviours: acquisition, intoxication, withdrawal, lifestyle Introduce behavioural strategies to reduce abusive and controlling behaviours
Lack understanding of range of impact of substance use on his thinking and behaviour	Psychological Capability	Knowledge	Education	Provide information on impact of substances on behaviours	Films demonstrating impact of substances via acquisition, intoxication, withdrawal, lifestyle and entitlement on behaviours
Poor ability to challenge Negative automatic thoughts (NATs) Poor perspective taking	Psychological Capability	Memory, attention and decision	Enablement	Advice on cues for conflict Elicit client input	Increase awareness of triggers/cues to relationship conflict Increase self-awareness
	Psychological Capability	Emotion	Persuasion Enablement	Show positive images Work ‘with’ clients’ motivation Highlight strengths	Film of masculinities to challenge Films of doing it differently to enhance motivation Focus on personal goals Develop skills to plan and enact positive relationship behaviours
	Physical opportunity	Context Resources	Enablement Education	Support non abusing partner Identify opportunities to practice	Proactive contact and information and support for non-abusing partner to minimise opportunity for ongoing abuse Value out of session work Provide additional telephone calls to encourage ‘try it out’ Teach positive relationship skills: perspective taking and communication
	Social opportunity	Social influences	Modelling	Demonstrate respectful communication	Show respectful positive communication between facilitators Respectful challenge between facilitators and group members Respect and ground rules in group

The APEASE criteria of Affordability, Practicability, Effectiveness and cost-effectiveness, Acceptability, Side effects/safety, and Equity considerations [46] were used in making context-based decisions on the content of interventions. The method here was also consultation and consensus. Thus, we selected the intervention functions that were possible to implement, linked with the evidence from previous empirical studies, and linked with clinical knowledge about what has been found to be effective with IPA and substance use populations.

From our research, education in IPA, substance use, and the interaction between these criteria and studies was key. Training and modelling would provide alternative strategies to interpret environmental cues, while enhancing self-regulation and distress tolerance and reducing the need for control within intimate relationships. Incentivisation was through offering a £5 voucher for every session attendance, which men would accumulate over the duration of the intervention for use in a pro social activity they chose (such as cinema tickets or restaurant vouchers). Vouchers are provided at session 6 and session 12. Persuasion to attend was identified as being helpful in promoting reflective and automatic motivation [39]. Enablement was envisaged as being delivered at a more structural level in terms of setting the intervention within a multi-disciplinary framework to work with the perpetrator to manage risk and to remove barriers to help seeking and promoting safety management for partners of the perpetrating men.

Based on the ‘what works’ body of knowledge from forensic psychology, the intervention was delivered in line with best practice for enhancing motivation and responsivity and was culturally competent, used active learning methods, visual and auditory materials. The intervention was manualised to maximise the integrity of the intervention. In line with the best practice guidelines of RESPECT, a UK non-Governmental domestic violence organisation, ADVANCE was designed as a group work intervention to facilitate peer challenge and maximise positive learning based on the zone of proximal learning; and reflecting the goal of enablement.

### Step 6: Identify policy categories

While the ADVANCE intervention focused mostly at the individual change level, it was delivered alongside proactive support, case management, and information sharing to manage risk and promote safety with partners and ex-partners of men in the group and was fully embedded within the justice, social services, and child protection systems structures to allow risk management and referral. This inclusion fits with best practice models [48, 49, 57, 58, 63] for IPA intervention developed from Duluth onwards and supported by RESPECT, and is in line with UK government policies, and reflects the outer ring of the BCW of using legislation, regulation, service provision, and guidelines to promote desired goals.

### Step 7: Select behaviour change techniques

**Fig. 2:**
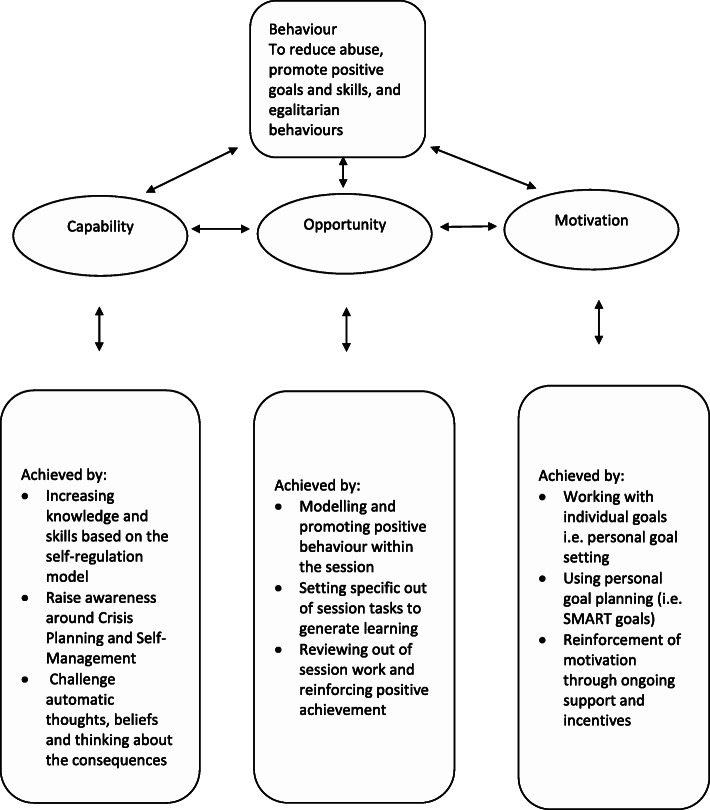
The ADVANCE model

Based on the BCW, Table 2 shows the specific behaviour change techniques (BCTs) linked to our formulation of the key elements underpinning change. BCTs are mapped to address each intervention function. The authors identified and agreed on those BCTs most practicable and effective in promoting behaviour change in men in substance use treatment who perpetrate IPA. In summary, for ADVANCE, we aimed to improve capability by the strategies described in Fig. 2.

### Step 8: Modes of delivery

Applying best practice from the ‘what works’ literature and RESPECT, the intervention was primarily face-to-face structured group work. Prior to group work, individual sessions assessed the client’s IPA, substance use, and motivation to change. Within groups, a range of modes was used: illustrative handouts of basic concepts, individual worksheets, and exercises conducted in pairs and small groups, role plays, between-session assignments, group’s discussion, presentations, and skills practice. A major innovation was the inclusion of video scenarios which were enactments of interactions derived from an amalgamation of individual stories within the dyad research [55] used as a focus for group discussion.

#### Nominal Group Technique

Following the initial identification of content and approach, a nominal group approach was implemented to refine the content in light of expert knowledge [13]. The nominal group technique took the form of 4 stages across a number of face-to-face meetings at which written records were kept and actions and revisions of materials were circulated after each meeting.

1. Initial themes drawn from the research and the systematic review identified key elements that needed to be included, e.g. CBT, integrated model, and strong motivational element, and identified topics to cover, e.g. including sexual jealousy and control, and the complex model of substance and IPV interactions and created an initial proposal which was discussed and refined by the team of experts from clinical psychology, forensic psychology, addictions, victim support, and IPV accreditation group.
2. Evidence from ‘what works’ literature, the Risk Needs Responsivity model and the Good Lives model, alongside the addictions literature, were then used to translate the topics into key targets for the intervention with proposed delivery approaches.
3. These key targets (Table 2) were then discussed and refined with input from experts and a proposed model with session content was developed.
4. The proposed model, session content, delivery style, and ethos were then created into programme manuals and materials which were reviewed by the expert group and then presented to the wider expert group through two learning alliances, at which feedback was noted, then a summary actions of what MUST be changed, what might be changed and what would not be changed were made, and then these changes were implemented to create the pilot intervention.

As the next stage in the ADVANCE programme was a pilot study focusing on feasibility and acceptability, there was no specific pilot conducted; however, patient-public consultation (PPI consultation) was undertaken with men and women how they had been affected by IPV to ensure the acceptability and clarity of the language, the acceptability of the content, and the acceptability within the pilot intervention.

#### PPI feasibility consultation

The PPI consultation involved 3 men and 5 women. The consultation took the form of presenting proposed content and with prompts, and noting the responses which were then collated to create summarises, which were then discussed as a team, and dominant threads of what could be adjusted without moving too far from the underpinning model incorporated. For example, initial plans for the intervention were initially ‘wordy’; however, following consultation, our examples and worksheets were revised to use more pictures and examples and less dense language.

### ADVANCE model

The ADVANCE intervention consisted of up to four pre-group individual sessions to assess and motivate participants. These sessions helped each participant consider their motivation for taking part, translated that into individual goals, and addressed any additional support needs to facilitate engagement, followed 12 × 2-h group sessions (see Table 3). The intervention was delivered by substance use service workers trained in its delivery. Key workers contacted participants by telephone between sessions to deal with problems and motivate individuals to attend the next session. Integrated Support Services (ISS) workers provided support to participants’ current/ex partner’s at least three times across the intervention period on their current/ex-partner’s attendance and progression, with the consent of the participants. ISS workers attended case management meetings with the group facilitators and substance use workers (approximately five times across the duration of the research) to ensure good communication and to manage any risk.

**Table 3:** ADVANCE sessions

Session title	Session objectives
1. Introduction	1. Get to know fellow group members 2. Understand the aims of the group 3. Understand what IPA is and how substance use can affect such behaviours 4. Learn new skills that can help in times of distress
2. Managing Myself	1. Shift focus from managing your relationship to managing yourself 2. Understand how substance use affects self-regulation 3. Be able to identify self-regulation and monitoring skills
3. Being a Respectful Man	1. Examine costs and pay-offs when being abusive 2. Identify triggering situations 3. Have improved self-awareness 4. Practise behavioural analysis
4. Impact of Intimate Partner Abuse	1. Understand the key aspects of IPA behaviours and how substance use affects them 2. Understand the impact of IPA on women 3. Continue to practise behaviour analysis
5. Children and Parenting	1. Recognise the impact of childhood experiences 2. Be able to identify the impact of witnessing IPA on children 3. Be able to identify the impact of parental substance use on children 4. Accept the past, build resilience, and learn from mistakes 5. Identify the strategies that lead to repeat or not repeat
6. Relating	1. Promote respectful and equal behaviours in ongoing relationships 2. Give up controlling behaviours within a relationship 3. Be able to recognise and challenge relationship jealousy 4. Become aware of unhelpful automatic thoughts and core beliefs
7. Improving Communication	1. Recognise challenges to communication in relationships and when using substances 2. Reduce abusive communication and increase respectful egalitarian communication 3. Develop a staying safe plan
8. Dealing with Distress	1. Understand what distress is 2. Learn to manage mood and emotions 3. Understand how substance use affects distress 4. Understand thinking errors and their impact
9. Planning to be Better	1. Identify high risk situations for IPA 2. Develop plans to manage high risk situations 3. Increase skills for staying safe
10. Positive Relationships	1. Understand the impact of behaviours in different relationships: substance using relationship, non-substance using partners, substance use discordant relationships 2. Be able to identify features and benefits of equal relationships 3. Be motivated and capable of using respectful behaviours in relationships
11. New Future, People’s Plans, Positive Activities	1. Create and engage with positive social networks 2. Identify meaningful activities and positive behaviours 3. Select realistic positive goals 4. Identify explicit positive life goals
12. Recap ‘What Have We Learned’	1. Describe new skills, identify strengths and progress 2. Identify positive resources to help maintain change 3. Identify further referrals 4. Understand where to reach help, support, follow up and to say goodbye

## Discussion

In developing the ADVANCE intervention, we accessed various theoretical models and frameworks to inform the content and approach. It used the BCW to translate this theoretical knowledge into specific targets for change and specific intervention approaches. In describing the approach and the resultant ADVANCE intervention, we have satisfied Hoddinott's [29] call for studies that describe the rationale, processes, and methods used in developing an intervention. Thus, we can claim that the ADVANCE intervention has been developed using a rigorous methodology, in particular the use of an explicit statement of the theory-based targets for change and of the appropriate mechanisms by which change should be supported. The systematic application of the BCW meant that the specific methods used were selected with reference to the type of change desired, namely improved capability, opportunity, or motivation.

This approach resulted in an intervention that met its key aim of addressing both IPA and substance use in an integrated fashion rather than addressing them as two separate problems. The intervention differed from other perpetrator programmes by offering specific knowledge and related skills that addressed both IPA and substance use in each session. It also incorporated other mainstream factors involved in IPA, namely masculine power, control, beliefs and attitudes, and aggression emanating from emotion dysregulation. It also explicitly used a multifaceted model of the range of links between the various aspects of substance use namely intoxication, withdrawal and physiological discomfort, drug seeking and acquisition, substance using lifestyle, and a gendered view of substance use.

The utility of applying the BCW model was the specificity and clarity brought to the intervention. Previous intervention development studies using the BCW state that the use of a ‘comprehensive supra-theory model’ [2] allowed the developers to access multi-factor models and more than one theory of change, for example, including use of incentives to encourage behaviour change based on behavioural principles alongside self-regulation such as effort regulation and attentional focus based on psychological principles. The challenges of applying this model were that it constrained the focus of the intervention to something that could be explicitly stated and potentially constrained the intervention developers from including more holistic targets, for example improvement on more global but less specific measures of well-being, life satisfaction or self-love as intervention goals.

### Strengths and limitations

This study strengthens the theoretical foundations on which to develop integrated interventions for IPA and substance to reduce or stop IPA. There has long been a call for integrated aggression and substance use interventions, yet true integration has been rare in interventions for general aggression [43] and for IPA [61]. One of the limitations is the difficulty in capturing each level of any factor that can contribute to IPA. By its very nature a programme focussing on individuals will over-emphasise the role of individual factors so there is a danger of minimising the ongoing need to also address societal and structural factors which could be problematic. Also, it is very difficult to know to what extent the observed factors associated with IPA contribute to different types of IPA (for instance physical violence versus coercive control) in different groups of perpetrators (for instance those of different ethnicity and different sexual orientation) in different contexts. Across this process, these issues have been addressed by taking common features and common pathways to identify possible routes and motives for IPA that should address the main features for a majority of substance using men within a UK context: it will not cover all needs and pathways and it is unlikely to address the needs of those from different cultures and contexts.

### Implications

The original intervention was refined following the PPI feasibility consultations, and the content of sessions honed and the delivery and training refined to produce a better more polished version of the original, but still in line with the BCW principles. This intervention development led onto a feasibility randomised controlled trial (RCT) among 104 male perpetrators attending substance use treatment in England. The full details are reported elsewhere [14, 24], but in brief this study identified that it was possible for trained substance use staff to deliver the ADVANCE intervention in substance use treatment services and that men who attended and staff who delivered the intervention evaluated it highly. Following this, a full-scale, multi-site randomised controlled trial to compare the effectiveness of the ADVANCE intervention plus substance use treatment as usual (TAU) to substance use TAU only, and a nested process evaluation will explore the ‘what works and how and for whom’ was planned. However due to Covid-19 restrictions, it was not possible to conduct the trial. The ADVANCE intervention has now been adapted for digital delivery and further feasibility testing is being undertaken. This will in turn help develop the theoretical understanding of what features are necessary and sufficient for IPA to occur in men who use substances in a UK context and refine knowledge of how best to target related risks to reduce incidence.

### Conclusion

IPA in substance use populations is high. Traditional interventions are not effective overall, and additionally those who are substance users are often screened out of generic interventions due to the need to address their substance use first. Using the BCW it was possible to systematically develop an integrated intervention based on what is known about IPA and substance use and making use of theoretically informed behaviour change mechanisms.

## Data Availability

The research data underpinning the materials described within this paper will be held in accordance with NIHR guidance and available for secondary analysis as agreed under the NIHR guidance and that of KCL, SLAM and in accordance with the ethical approval granted.

## Footnotes

1 While WHO uses IPV as a current inclusive term for domestic abuse, we use the term intimate partner abuse (IPA) to avoid focusing on physical violence, which we feel better reflects an ongoing pervasive pattern of abuse and avoids focusing on individual incidents.
